# Beyond Proxies: Environment, Neighborhood, and Racial Disparities in Hypertensive Disorders of Pregnancy

**DOI:** 10.1016/j.focus.2026.100493

**Published:** 2026-03-23

**Authors:** Noreen Singh, Jacqueline M. Chiofalo, Saskia Z. Shuman, Aimee L. Smith

**Affiliations:** 1 The Institute for Family Health, New York, New York; 2 Mathematica, Princeton, New Jersey; 3 The Institute for Family Health, Kingston, New York

**Keywords:** Preeclampsia, gestational hypertension, environmental exposure, neighborhood poverty, residential segregation, structural determinants

## Abstract

**Introduction:**

Hypertensive disorders of pregnancy are leading causes of maternal morbidity and mortality in the U.S., with persistent racial disparities. Current clinical guidelines include Black race and lower income as proxy risk factors, which lack precision and may obscure structural and environmental contributors to hypertensive disorders of pregnancy risk. This study evaluated environmental and neighborhood-level exposures as clinically accessible, structurally grounded risk factors for hypertensive disorders of pregnancy that can be feasibly implemented in practice.

**Methods:**

A retrospective cohort study was conducted among 619 pregnant patients receiving care within a Federally Qualified Health Center network. The primary outcome was hypertensive disorders of pregnancy, defined as gestational hypertension or preeclampsia diagnosed during pregnancy, intrapartum, or postpartum. Address-linked exposures included high nitrogen dioxide pollution (≥5 ppb), high neighborhood poverty (>30% of residents below the federal poverty level), and ZIP code–level racial composition (majority White and majority Black or Hispanic ZIP codes). Associations with hypertensive disorders of pregnancy were assessed in the full cohort and in race-stratified models, with and without adjustment for aspirin prescription. ORs with 95% CIs were reported.

**Results:**

Among 619 patients, 114 (18.4%) developed hypertensive disorders of pregnancy. High nitrogen dioxide pollution was associated with the highest odds of hypertensive disorders of pregnancy (OR=3.73, 95% CI=2.43, 5.79, *p*<0.001), followed by high neighborhood poverty (OR=2.21, 95% CI=1.45, 3.43, *p*<0.001). After adjustment for aspirin prescription, high nitrogen dioxide exposure remained the strongest risk factor (OR=2.88, 95% CI=1.81, 4.61, *p*<0.001). Residence in majority White ZIP codes was protective for both Black (OR=0.41, 95% CI=0.20, 0.81, *p*=0.012) and non-Black (OR=0.37, 95% CI=0.22, 0.64, *p*<0.001) individuals.

**Conclusions:**

High nitrogen dioxide pollution exposure and high neighborhood poverty were independently associated with increased hypertensive disorders of pregnancy risk across racial groups, with nitrogen dioxide demonstrating the strongest association and effect size comparable with that of established clinical high-risk factors. Environmental and neighborhood-level measures may enhance hypertensive disorders of pregnancy risk assessment beyond race- and income-based proxies and can be derived from routinely available address-linked data.

## HIGHLIGHTS

• High nitrogen dioxide (≥5 ppb) showed the strongest association with hypertensive disorders of pregnancy (HDP).
• High neighborhood poverty (>30%) was associated with increased HDP risk.
• Majority White ZIP codes were protective across racial groups.
• Environmental associations persisted after adjustment for aspirin prescription.
• Address-linked area-level measures strengthen HDP risk assessment beyond proxies.

## INTRODUCTION

Hypertensive disorders of pregnancy (HDP) are leading causes of severe maternal morbidity and mortality in the U.S., with increasing prevalence and persistent racial disparities.^1^ Current guidelines from the American College of Obstetricians and Gynecologists (ACOG) and the U.S. Preventive Services Task Force (USPSTF) address these disparities by including Black race and lower income as risk factors for preeclampsia.^2^^,^^3^ Although these guidelines acknowledge that Black race serves as a proxy for underlying structural racism rather than biological propensity,^4^ this approach falls short of providing actionable guidance and precise definitions that can be used in clinical risk assessment models. Using race-based risk factors may also inadvertently reinforce bias and obscure the structural and environmental determinants of HDP risk.^5^^,^^6^ Moreover, the lack of a clear definition for lower income limits the effectiveness of current risk stratification and intervention efforts.

A growing body of literature has demonstrated that environmental and neighborhood-level exposures are important drivers of maternal health disparities.^7^^,^^8^ Black and other racial/ethnic minority populations in the U.S. face disproportionately higher exposures to environmental pollutants such as nitrogen dioxide (NO₂) owing to historical disinvestment, redlining, and industrial zoning.^9^, ^10^, ^11^ NO₂ was selected as the primary air pollution marker in this study given its accessibility and established relevance in the epidemiologic literature on HDP.^12^^,^^13^ Although fine particulate matter (PM2.5) is also strongly implicated in HDP risk, its exposure assessment typically requires integration of ground-based monitors, satellite data, and atmospheric models that are not consistently available.^14^ NO₂ has demonstrated independent effects on cardiovascular and pregnancy outcomes, even after adjustment for copollutants, with meta-analyses and large cohort studies showing significant associations between maternal NO₂ exposure and increased risk of HDP, preeclampsia, and adverse birth outcomes.^7^^,^^12^^,^^13^^,^^15^^,^^16^ Collectively, these findings support the use of NO₂ as a practical and scientifically valid indicator for assessing environmental contributions to maternal health disparities.

Building on this environmental framework, neighborhood context provides a critical lens for understanding how structural inequities shape maternal health. Neighborhood racial composition should be conceptualized not as a harmful exposure itself but as a proxy for residential segregation and structural racism. Segregation, rooted in policies such as redlining, exclusionary zoning, and sustained disinvestment, has concentrated environmental hazards and limited access to health-promoting resources in predominantly Black communities. Accumulating evidence indicates that these structural and place-based inequities contribute to elevated risks of HDP, preterm birth, and severe maternal morbidity, even after accounting for individual-level factors.^17^, ^18^, ^19^, ^20^ Longitudinal studies show that Black birthing people living in neighborhoods characterized by concentrated racial and economic segregation have significantly higher odds of HDP than those in more privileged areas, independent of personal SES. Neighborhood poverty further amplifies these effects, with residence in high poverty, segregated areas consistently associated with greater HDP risk.^18^^,^^21^ Together, these findings highlight the need to reorient HDP clinical risk assessment toward neighborhood and environmental conditions, such as neighborhood poverty and segregation, in place of race-based proxies.

Accordingly, this study expands upon prior work by incorporating environmental and neighborhood-level exposures as measurable structural determinants of HDP. The objective was to identify clinically accessible, equity-informed risk factors that can be feasibly implemented in practice. By leveraging routinely available data such as patient addresses, patients were linked to neighborhood-level indicators of racial composition, poverty, and NO₂ exposure to refine HDP risk assessment. This approach addresses key limitations of current race- and income-based guidelines, which are neither granular nor easily implementable in clinical settings. For example, although nearly all patients in the Federally Qualified Health Center network live below 100% of the federal poverty level, only 46.2% had individual poverty data available in 2024, making low income an impractical risk factor for systematic use. Incorporating neighborhood- and environmental indicators provides a more precise and replicable means to capture structural and environmental context. Ultimately, this work seeks to inform more equitable and actionable HDP risk stratification strategies, consistent with current society guidelines calling for the integration of social and environmental determinants into maternal health research and practice.^17^^,^^22^

## METHODS

This study was approved by the IRB at the Institute for Family Health.

### Study Sample

A retrospective cohort study was conducted using chart review of the electronic health record (EHR) with complete data for all variables of interest (*n*=619) among pregnant individuals aged ≥18 years who received 2 or more prenatal visits at any 1 of 24 full-time sites in a Federally Qualified Health Center network in New York State between January 1, 2019 and December 31, 2020. Study data were collected and managed using REDCap. Initially, all pregnant individuals who met inclusion criteria between 2019 and 2020 were identified to begin chart review. However, because manual review was resource intensive and the baseline prevalence of HDP is relatively low in unselected populations, sampling shifted to purposive sampling, targeting patients with documented ACOG risk factors for preeclampsia to ensure adequate statistical power within risk categories. This approach maximized efficiency while focusing on a clinically relevant high-risk population most likely to benefit from enhanced risk stratification. The 18.4% HDP prevalence in the sample reflects this purposive sampling of patients with ACOG risk factors, because population-based studies typically report HDP prevalence of 5%–8% in unselected pregnant populations, whereas rates up to 15%–25% are expected in high-risk cohorts with multiple risk factors.^23^, ^24^, ^25^

### Measures

Variables examined include ACOG risk factors: age and parity at the time of delivery, race, granular ethnicity (self-reported by patients), insurance type, prepregnancy BMI, documented prior history (preeclampsia, chronic hypertension, diabetes mellitus, chronic kidney disease, autoimmune disease, preterm birth, low birth weight/small for gestational age infant, current multifetal gestation, interpregnancy interval greater than 10 years), and family history of preeclampsia.^2^^,^^3^ Perinatal outcomes examined include gestational age at delivery, delivery type, birth weight, and diagnosis of preeclampsia or gestational hypertension. HDP diagnoses were identified using multiple data sources, including hospital discharge summaries (when available), outpatient postpartum visit notes, International Classification of Diseases codes, and EHR problem lists. Race was treated as a social classification as recorded in the EHR; Black is used in the remaining parts of this paper as shorthand for the Black/African American category.

Environmental and neighborhood-level exposures were defined within a structural determinants framework, examining how place-based conditions shaped by historical policies and contemporary practices contribute to HDP risk independent of individual characteristics. On the basis of other work with high-risk populations, high NO₂ exposure was defined as ≥5 ppb, aligning with evidence that adverse health outcomes, including increased mortality, can occur at even modest NO₂ concentrations.^26^, ^27^, ^28^ Neighborhoods with more than 30% of residents below the federal poverty line were categorized as high poverty, supported by prior epidemiologic studies and consistent with federal definitions of concentrated poverty.^29^, ^30^, ^31^ Environmental variables were created to classify patients by high versus low NO₂ pollution, majority Black or Hispanic ZIP code, majority White ZIP code, and ZIP code poverty rate (>30% versus ≤30% of residents below the poverty level). Neighborhood racial composition variables were derived from U.S. Census categories and were analyzed as area-level measures distinct from individual race/ethnicity. Binary variables of the ACOG risk factors were created for analysis.

### Statistical Analysis

Patient addresses during prenatal care were collected from the EHR and subsequently geocoded. These geocoded addresses were linked to U.S. Census American Community Survey 5-year estimates (2015–2019) to derive ZIP code–level demographic and poverty data. NO₂ pollution data were obtained from the University at Albany, State University of New York, and the University of Wisconsin-Madison Institute for Environmental Studies, representing annual average concentrations. Environmental exposure variables were created using qGIS spatial analysis software to assign exposure levels on the basis of residential ZIP codes during pregnancy.

The outcome of interest was HDP, defined as a diagnosis of preeclampsia or gestational hypertension during pregnancy, intrapartum, or postpartum. Univariate logistic regression models were created for each risk factor and outcome. To account for evidence-based prophylactic strategies for HDP, a second model was created adjusting for aspirin prescription during the pregnancy. If there was documentation of a provider’s recommendation for low-dose aspirin (81 mg daily) or an aspirin prescription in the patient’s electronic medical record during the pregnancy, the patient was deemed to have been prescribed low-dose aspirin. Models were adjusted only for aspirin prescription, because low-dose aspirin represents the primary and most widely recommended intervention for HDP prevention in at-risk pregnancies, endorsed by ACOG, the Society for Maternal-Fetal Medicine, and the USPSTF.^2^^,^^3^ Given its established efficacy and widespread guideline endorsement, adjusting for aspirin prescription allowed evaluation of the independent associations of environmental and neighborhood-level exposures with HDP risk beyond current preventive care.

For individual-level stratified analyses, race was operationalized as Black/African American versus non-Black to align with clinical guideline use of Black race as a proxy risk factor for preeclampsia and with well-documented inequities in HDP linked to structural racism. The non-Black category included White, Hispanic, Asian, and other racial/ethnic groups. Race-stratified analyses were conducted using separate logistic regression models rather than interaction terms to facilitate clearer interpretation of race-stratified effects. The final study size was determined by the number of patient charts that could be manually reviewed during the study period that met inclusion criteria and had complete data for all variables of interest. Prior to analysis, all records with missing data for any exposure, covariate, or outcome variables were excluded to ensure consistency across regression models. Data analysis was performed using RStudio.

## RESULTS

Table 1 presents the characteristics of patients diagnosed with HDP, including environmental risk factors examined in this study and established high-risk and moderate-risk factors as defined by ACOG and USPSTF. Of the 619 patients included in the cohort, 114 (18.4%) were diagnosed with HDP. The mean age of the study population was 28.8 years, with the HDP group slightly older (29.1 years) than the non-HDP group (28.7 years). The mean prepregnancy BMI was higher in the HDP group (30.5) than in the non-HDP group (28.5). Aspirin therapy was prescribed in 18.3% of all patients, with a notably higher proportion in the HDP group (36.8%) than in the non-HDP group (14.1%). Black patients comprised 29.4% of the study population, with a higher representation in the HDP group (43.0%) than in the non-HDP group (26.3%).

**Table 1:** Characteristics of Patients by Diagnosis of Hypertensive Disorders of Pregnancy

Characteristic	Category	No diagnosis of HDP (*n*=505)	Diagnosis of HDPᵃ (*n*=114)	Total (*n*=619)
Age, years	Mean (SD)	28.7 (6.03)	29.1 (6.07)	28.8 (6.04)
	Median (minimum, maximum)	28.0 (18.0, 44.0)	28.5 (19.0, 45.0)	28.0 (18.0, 45.0)
Aspirin therapy initiated during pregnancy	No	434 (85.9%)	72 (63.2%)	506 (81.7%)
	Yes	71 (14.1%)	42 (36.8%)	113 (18.3%)
Environmental factors				
Majority Black or Hispanic ZIP code	No	274 (54.3%)	51 (44.7%)	325 (52.5%)
	Yes	231 (45.7%)	63 (55.3%)	294 (47.5%)
Majority White ZIP code	No	157 (31.1%)	65 (57.0%)	222 (35.9%)
	Yes	348 (68.9%)	49 (43.0%)	397 (64.1%)
Percentage poverty in ZIP code	≤30%	255 (50.5%)	36 (31.6%)	291 (47.0%)
	>30%	250 (49.5%)	78 (68.4%)	328 (53.0%)
High NO₂ pollution area	NO_2_ <5	324 (64.2%)	37 (32.5%)	361 (58.3%)
	NO_2_ ≥5	181 (35.8%)	77 (67.5%)	258 (41.7%)
High risk factors				
History of preeclampsia	No	483 (95.6%)	101 (88.6%)	584 (94.3%)
	Yes	22 (4.4%)	13 (11.4%)	35 (5.7%)
History of chronic hypertension	No	488 (96.6%)	98 (86.0%)	586 (94.7%)
	Yes	17 (3.4%)	16 (14.0%)	33 (5.3%)
History of diabetes	No	486 (96.2%)	110 (96.5%)	596 (96.3%)
	Yes	19 (3.8%)	4 (3.5%)	23 (3.7%)
History of chronic kidney disease	No	502 (99.4%)	113 (99.1%)	615 (99.4%)
	Yes	3 (0.6%)	1 (0.9%)	4 (0.6%)
History of autoimmune disease	No	495 (98.0%)	111 (97.4%)	606 (97.9%)
	Yes	10 (2.0%)	3 (2.6%)	13 (2.1%)
More than one fetus during pregnancy	No	499 (98.8%)	114 (100%)	613 (99.0%)
	Yes	6 (1.2%)	0 (0%)	6 (1.0%)
Moderate risk factors				
Black/African American	No	372 (73.7%)	65 (57.0%)	437 (70.6%)
	Yes	133 (26.3%)	49 (43.0%)	182 (29.4%)
Advanced maternal age	No	407 (80.6%)	88 (77.2%)	495 (80.0%)
	Yes	98 (19.4%)	26 (22.8%)	124 (20.0%)
BMI ≥30	No	344 (68.1%)	59 (51.8%)	403 (65.1%)
	Yes	161 (31.9%)	55 (48.2%)	216 (34.9%)
Family history of preeclampsia	No	501 (99.2%)	112 (98.2%)	613 (99.0%)
	Yes	4 (0.8%)	2 (1.8%)	6 (1.0%)
Interpregnancy interval >10 years	No	486 (96.2%)	108 (94.7%)	594 (96.0%)
	Yes	19 (3.8%)	6 (5.3%)	25 (4.0%)
Nulliparous	No	426 (84.4%)	80 (70.2%)	506 (81.7%)
	Yes	79 (15.6%)	34 (29.8%)	113 (18.3%)

^a^In each column, *n* refers to the number of patients without a diagnosis of HDP (*n*=505) and with a diagnosis of HDP (*n*=114). Percentages in each column are calculated on the basis of the total population within the respective subgroup.

^b^Owing to census data for race and ethnicity being self-reported, multiple-selection entry, sums will not add up to 100%.

^c^The reference group for majority Black or Hispanic neighborhood includes all neighborhoods that are not majority Black or Hispanic (i.e., mixed or majority White).

HDP, hypertensive disorders of pregnancy; NO_2_, nitrogen dioxide.

Regarding environmental factors, 47.5% of patients in this sample lived in majority Black or Hispanic ZIP codes, with a higher proportion in the HDP group (55.3%) than in the non-HDP group (45.7%). Conversely, 64.1% lived in majority White ZIP codes, with a lower proportion in the HDP group (43.0%) than in the non-HDP group (68.9%). High poverty ZIP codes (>30% poverty rate) were home to 53.0% of patients, with a higher proportion in the HDP group (68.4%) than in the non-HDP group (49.5%). Similarly, 41.7% lived in high pollution areas (NO₂ ≥5 ppb), with a markedly higher proportion in the HDP group (67.5%) than in the non-HDP group (35.8%).

**Figure 1:**
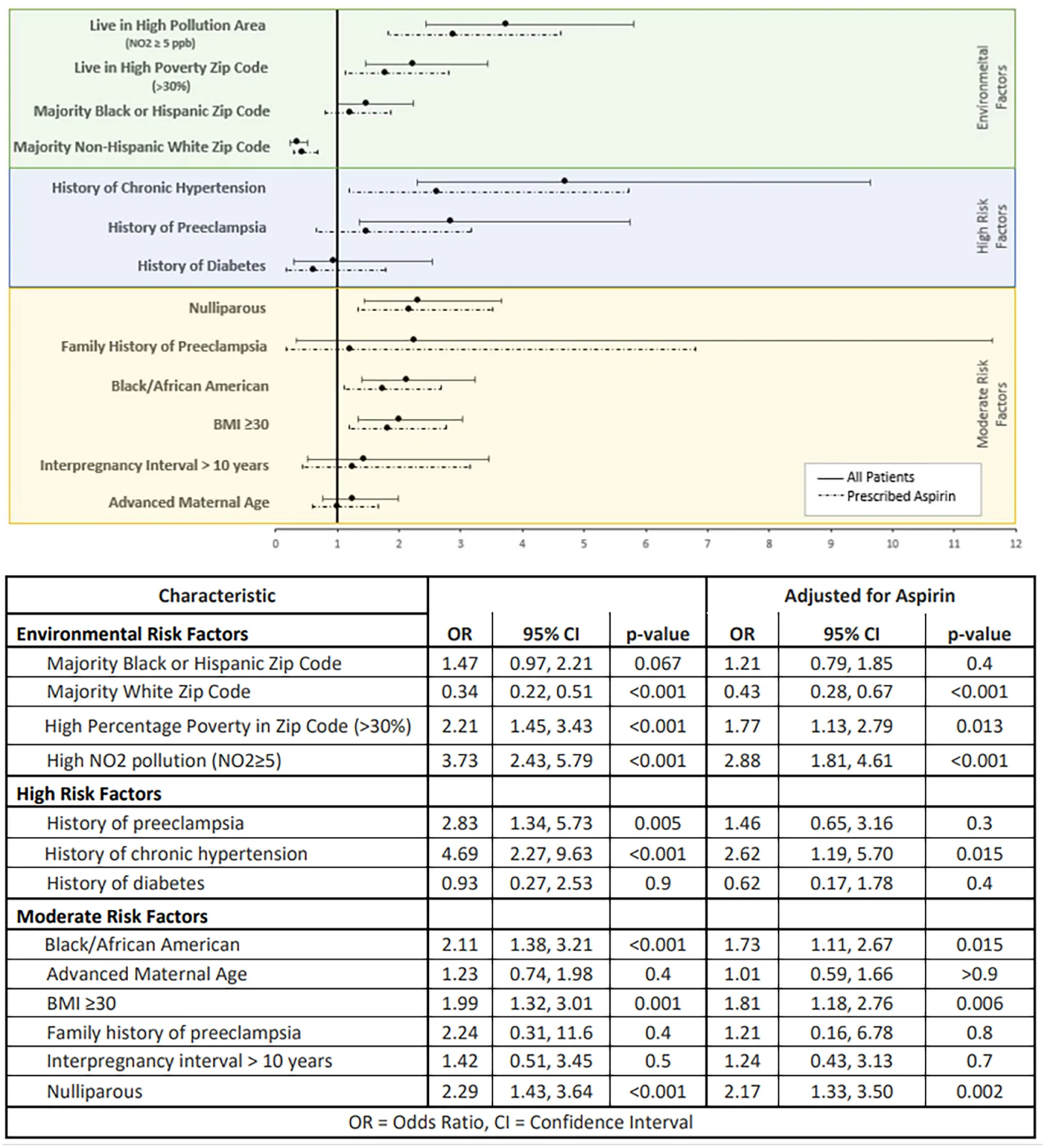
ORs for environmental and clinical risk factors associated with hypertensive disorders of pregnancy.

OR analyses showed significant associations between several environmental exposures and risk of HDP. Figure 1 presents a forest plot and corresponding table of unadjusted and aspirin-adjusted ORs for environmental exposures (green) alongside established high-risk (blue) and moderate-risk (yellow) clinical factors defined by ACOG and USPSTF. In unadjusted analyses, living in a high NO₂ pollution area (NO₂ ≥5 ppb compared with NO₂ <5 ppb) was associated with the highest odds of HDP (OR=3.73, 95% CI=2.43, 5.79, *p*<0.001), followed by living in a high-poverty (>30%) ZIP code (OR=2.21, 95% CI=1.45, 3.43, *p*<0.001) compared with living in a low-poverty ZIP code. Living in a majority White ZIP code was protective (OR=0.34, 95% CI=0.22, 0.51, *p*<0.001). After adjustment for aspirin prescription, the primary evidence-based intervention for HDP prevention, these environmental associations remained robust. High NO₂ pollution areas continued to show the strongest association with HDP (AOR=2.88, 95% CI=1.81, 4.61, *p*<0.001), followed by high poverty ZIP codes (AOR=1.77, 95% CI=1.13, 2.79, *p*=0.013). Living in a majority White ZIP code remained protective (AOR=0.43, 95% CI=0.28, 0.67, *p*<0.001).

Among established high-risk factors defined by ACOG, a history of chronic hypertension showed the strongest unadjusted association with HDP (OR=4.69, 95% CI=2.27, 9.63, *p*<0.001), followed by a history of preeclampsia (OR=2.83, 95% CI=1.34, 5.73, *p*=0.005). Moderate-risk factors in unadjusted analyses included nulliparity (OR=2.29, 95% CI=1.43, 3.64, *p*<0.001), Black race (OR=2.11, 95% CI=1.38, 3.21, *p*<0.001), and prepregnancy BMI ≥30 (OR=1.99, 95% CI=1.32, 3.01, *p*=0.001). After adjusting for aspirin prescription, established high-risk factors showed attenuated but variable associations: chronic hypertension remained significant (AOR=2.62, 95% CI=1.19, 5.70, *p*=0.015), whereas history of preeclampsia was no longer significant (AOR=1.46, 95% CI=0.65, 3.16, *p*=0.3). Among moderate-risk factors, aspirin-adjusted associations were nulliparity (AOR=2.17, 95% CI=1.33, 3.50, *p*=0.002), Black race (AOR=1.73, 95% CI=1.11, 2.67, *p*=0.015), and BMI ≥30 (AOR=1.81, 95% CI=1.18, 2.76, *p*=0.006). After aspirin adjustment, high NO₂ pollution exposure showed the highest adjusted OR among all risk factors (AOR=2.88, 95% CI=1.81, 4.61, *p*<0.001), with an effect similar in magnitude to that of chronic hypertension (AOR=2.62, 95% CI=1.19, 5.70, *p*=0.015).

Table 2 presents the OR associations between environmental risk factors and HDP diagnosis, stratified by race and adjusted for aspirin prescription. In predominantly Black or Hispanic ZIP codes, Black individuals showed no significant association with HDP risk (OR=0.81, 95% CI=0.42, 1.56, *p*=0.5), whereas non-Black individuals in these ZIP codes had significantly higher odds of developing HDP (OR=1.95, 95% CI=1.15, 3.35, *p*=0.014). In predominantly White ZIP codes, both Black and non-Black individuals had significantly lower odds of developing HDP (OR=0.41, 95% CI=0.20, 0.81, *p*=0.012 and OR=0.37, 95% CI=0.22, 0.64, *p*<0.001, respectively). In areas with high NO₂ pollution (≥5 ppb), both Black (OR=3.4, 95% CI=1.54, 8.32, *p*=0.004) and non-Black (OR=3.33, 95% CI=1.95, 5.75, *p*<0.001) individuals had significantly higher odds of developing HDP.

**Table 2:** ORs for Environmental and Neighborhood Exposures and HDP, Stratified by Race, With and Without Aspirin Adjustment

Environmental risk factors	Black/African American patients			Black/African American patients prescribed aspirin			White patients			White patients prescribed aspirin		
	OR	95% CI	*p*-value	OR	95% CI	*p*-value	OR	95% CI	*p*-value	OR	95% CI	*p*-value
Majority White ZIP code	0.41	0.2, 0.81	0.012	0.45	0.22, 0.92	0.031	0.37	0.22, 0.64	<0.001	0.5	0.28, 0.9	0.019
Majority Black or Hispanic ZIP code	0.81	0.42, 1.56	0.5	0.68	0.34, 1.35	0.3	1.95	1.15, 3.35	0.014	1.66	0.95, 2.9	0.073
High percentage poverty in ZIP code (>30%)	2.01	1, 4.26	0.057	1.78	0.87, 3.83	0.12	2.1	1.23, 3.68	0.008	1.63	0.92, 2.92	0.1
High NO_2_ pollution area (NO_2_ ≥5 ppb)	3.4	1.54, 8.32	0.004	2.91	1.28, 7.33	0.015	3.33	1.95, 5.75	<0.001	2.45	1.35, 4.41	0.003

HDP, hypertensive disorders of pregnancy; NO_2_, nitrogen dioxide.

Table 3 presents the OR associations for all risk factors and HDP diagnosis, stratified by patient race and adjusted for aspirin prescription. For Black patients, living in a high pollution area was associated with the highest odds of HDP (OR=3.4, 95% CI=1.54, 8.32, *p*=0.004) across environmental and ACOG high- and moderate-risk factors, whereas living in a majority White ZIP code was associated with the lowest odds of HDP (OR=0.41, 95% CI=0.20, 0.81, *p*=0.012). Among non-Black individuals, history of chronic hypertension was associated with the highest odds of HDP (OR=6.39, 95% CI=2.27, 18.0, *p*<0.001) across all factors, followed by living in a high pollution area (OR=3.33, 95% CI=1.95, 5.75, *p*<0.001), whereas living in a majority White ZIP code was associated with the lowest odds of HDP (OR=0.37, 95% CI=0.22, 0.64, *p*<0.001). After adjustment for aspirin prescription, associations were attenuated but remained significant for most factors, particularly for high-pollution areas across both racial groups. Notably, for Black individuals, the association with high poverty areas became nonsignificant after aspirin adjustment (AOR=1.78, 95% CI=0.87, 3.83, *p*=0.12), whereas for non-Black individuals, the association with majority Black or Hispanic ZIP codes became marginally significant (AOR=1.66, 95% CI=0.95, 2.90, *p*=0.073).

**Table 3:** ORs for Environmental and Clinical HDP Risk Factors, Stratified by Race, With and Without Aspirin Adjustment

	Stratified for Black/African American patients						Stratified for non-Black/African American patients					
				Adjusted for Aspirin						Adjusted for Aspirin		
Characteristic	OR	95% CI	*p*-value	OR	95% CI	*p*-value	OR	95% CI	*p*-value	OR	95% CI	*p*-value
Environmental risk factors												
Majority Black or Hispanic ZIP code	0.81	0.42, 1.56	0.5	0.68	0.34, 1.35	0.3	2	1.15, 3.35	0.014	1.7	0.95, 2.90	0.073
Majority White ZIP code	0.41	0.20, 0.81	0.012	0.45	0.22, 0.92	0.031	0.4	0.22, 0.64	<0.001	0.5	0.28, 0.90	0.019
High percentage poverty in ZIP code (>30%)	2.01	1.00, 4.26	0.057	1.78	0.87, 3.83	0.12	2.1	1.23, 3.68	0.008	1.6	0.92, 2.92	0.1
High NO_2_ pollution (NO_2_ ≥5 ppb)	3.4	1.54, 8.32	0.004	2.91	1.28, 7.33	0.015	3.3	1.95, 5.75	<0.001	2.5	1.35, 4.41	0.003
High risk factors												
History of preeclampsia	2.6	0.87, 7.69	0.08	1.77	0.54, 5.68	0.3	2.6	0.89, 6.76	0.06	1.3	0.40, 3.61	0.7
History of chronic hypertension	2.69	0.95, 7.49	0.056	1.9	0.62, 5.72	0.3	6.4	2.27, 18.0	<0.001	3.5	1.15, 10.7	0.024
History of diabetes	0.53	0.03, 3.42	0.6	0.33	0.02, 2.25	0.3	1.2	0.28, 3.93	0.7	0.9	0.20, 3.09	0.9
Moderate risk factors												
Advanced maternal age	1.23	0.52, 2.75	0.6	1.06	0.44, 2.43	0.9	1.3	0.68, 2.36	0.4	1	0.52, 1.94	>0.9
BMI ≥30^a^	2	1.03, 3.91	0.04	1.94	0.99, 3.83	0.053	1.9	1.07, 3.15	0.025	1.6	0.93, 2.83	0.086
Family history of preeclampsia	1.84	0.24, 11.5	0.5	1.26	0.16, 8.20	0.8	0		>0.9	0		>0.9
Interpregnancy interval >10 years	0		>0.9	0		>0.9	3.1	1.03, 8.18	0.032	2.3	0.74, 6.58	0.13
Nulliparous	3.2	1.53, 6.69	0.002	3.18	1.50, 6.73	0.002	1.7	0.85, 3.09	0.12	1.5	0.77, 2.92	0.2

a Compared with reference group of average/normal BMI. HDP, hypertensive disorders of pregnancy; NO_2_, nitrogen dioxide.

## DISCUSSION

This study aimed to move beyond race- and low-income based proxy measures by evaluating environmental and neighborhood-level exposures that may serve as clinically accessible, structurally grounded risk factors for HDP. Findings demonstrate that living in areas with high neighborhood poverty (>30%) and high NO₂ pollution (≥5 ppb) was independently associated with significantly increased HDP risk across racial groups. Among all environmental variables examined, high NO₂ pollution showed the strongest association with HDP after aspirin adjustment, with an effect size comparable with that of established clinical high-risk factors.

Residence in predominantly White ZIP codes was protective and was associated with significantly lower HDP risk for both Black and non-Black individuals. This pattern likely reflects structural racism and residential segregation that have driven inequities in neighborhood investment and environmental burden, disproportionately affecting predominantly Black communities and shaping access to health-promoting resources such as clean air and safe housing.^17^^,^^18^^,^^32^

This analysis of environmental and socioeconomic conditions responds to recent calls in public health research to identify underlying structural drivers of racial disparities rather than simply documenting them and relying on race itself as a risk marker.^33^^,^^34^ By establishing that pollution and poverty contribute to HDP risk independent of individual race, the study highlights neighborhood conditions as important pathways through which structural inequities influence pregnancy outcomes. These observations build on prior research showing that neighborhood indices predict outcomes such as mortality, low birth weight, and HDP, even after accounting for individual socioeconomic factors.^17^^,^^18^^,^^35^

A notable finding is that NO₂ exposure remained a dominant risk factor for HDP even after adjustment for aspirin prescription. Although low-dose aspirin modestly reduces preeclampsia risk, it does not fully offset the oxidative stress, vascular dysfunction, or placental inflammation linked to chronic air pollution exposure.^36^^,^^37^ This aligns with recent systematic reviews demonstrating that air pollution, including NO₂, is a consistent and independent risk factor for HDP and adverse pregnancy outcomes, regardless of other clinical interventions.^7^^,^^12^^,^^13^ The persistence of HDP risk in high NO₂ areas despite aspirin prophylaxis underscores the limits of individual-level strategies and the need for population-level approaches addressing environmental exposures.

The relationship between neighborhood poverty and HDP risk observed in this study reflects a complex interplay of structural and individual factors. Although residence in high poverty neighborhoods was associated with increased HDP risk overall, the ORs for Black and non-Black individuals were similar with overlapping CIs, indicating no statistically meaningful difference between groups. This supports the interpretation that neighborhood poverty is an important contextual factor across populations and operates within broader pathways shaped by segregation and other structural influences.

Although high NO₂ exposure increased HDP risk consistently across racial groups, the relative contribution of traditional ACOG risk factors varied descriptively, with nulliparity appearing more influential among Black patients and chronic hypertension appearing more influential among non-Black patients. These differences are exploratory but suggest that environmental and clinical determinants likely act in combination rather than in isolation in shaping HDP risk. Together, these observations highlight the need for future research to more fully examine how neighborhood context interacts with individual clinical factors to influence HDP risk.

### Limitations

Several limitations of this study should be acknowledged. Aspirin adherence could not be confirmed among patients who received a prescription, and aspirin use could not be determined among patients without a prescription. Adjustment was limited to aspirin rather than multiple potential confounders, which limits causal inference but aligns with the objective of identifying clinically actionable risk factors that could enhance existing prediction models. Given the 18.4% prevalence of HDP in the study sample, the reported ORs may overestimate the corresponding risk ratios. Log-binomial regression models were fitted to estimate risk ratios directly; however, convergence failures occurred with several covariates, a common challenge with this approach. Therefore, the reported estimates should be interpreted as measures of association strength rather than precise RR estimates. Future studies with larger sample sizes should consider modified Poisson regression or other robust approaches to estimate risk ratios directly.

The use of ZIP code–level data may obscure within-neighborhood variation in pollution or socioeconomic conditions. Duration of residence and cumulative historical exposure to environmental hazards could not be assessed. Given that much of the study period occurred during 2020, when coronavirus disease 2019 (COVID-19) restrictions in New York City led to reduced traffic volumes, NO₂ measurements likely underestimate typical pollution exposure levels. Although NO₂ is a key marker of traffic-related air pollution, other pollutants may also contribute to HDP risk. In addition, although neighborhood racial composition and poverty are practical for clinical use, these measures cannot fully capture the cumulative impact of neighborhood deprivation. Consistent with the emphasis on clinical utility in a high-risk population, analyses aimed at causal inference or mediation were not conducted, such as examining dose–response relationships between NO₂ levels or poverty gradients and HDP outcomes. These represent important directions for future research.

## CONCLUSIONS

These findings have important implications for clinical practice and public health policy. Incorporating environmental and neighborhood-level data into HDP risk assessments, potentially through integration of census and ZIP code data into EHRs, may improve identification of at-risk individuals. Although aspirin prophylaxis remains a recommended preventive measure, sustainable reductions in HDP risk will require broader policy efforts addressing environmental exposures and neighborhood investment, particularly in communities facing high pollution and persistent disinvestment. A multilevel approach that combines clinical care with environmental and structural interventions may offer a more effective path toward reducing racial and socioeconomic disparities in HDP.
